# Differences in the Central Energy Metabolism of Cancer Cells between Conventional 2D and Novel 3D Culture Systems

**DOI:** 10.3390/ijms22041805

**Published:** 2021-02-11

**Authors:** Ryo Ikari, Ken-ichi Mukaisho, Susumu Kageyama, Masayuki Nagasawa, Shigehisa Kubota, Takahisa Nakayama, Shoko Murakami, Naoko Taniura, Hiroyuki Tanaka, Ryoji P. Kushima, Akihiro Kawauchi

**Affiliations:** 1 Department of Urology, Shiga University of Medical Science, Otsu 520-2192, Japan; ikariryo@belle.shiga-med.ac.jp (R.I.); kageyama@belle.shiga-med.ac.jp (S.K.); mn@belle.shiga-med.ac.jp (M.N.); kubota@belle.shiga-med.ac.jp (S.K.); kawauchi@belle.shiga-med.ac.jp (A.K.); 2 Division of Human Pathology, Department of Pathology, Shiga University of Medical Science, Otsu 520-2192, Japan; 330601@belle.shiga-med.ac.jp (T.N.); ntaniura@belle.shiga-med.ac.jp (N.T.); kushima@belle.shiga-med.ac.jp (R.P.K.); 3 Department of Oral and Maxillofacial Surgery, Shiga University of Medical Science, Otsu 520-2192, Japan; takamori@belle.shiga-med.ac.jp; 4 Department of Biochemistry and Molecular Biology, Shiga University of Medical Science, Otsu 520-2192, Japan; tanakah@belle.shiga-med.ac.jp

**Keywords:** cancer metabolism, three-dimensional, tissueoid cell culture system, urinary bladder cancer, prostate cancer

## Abstract

The conventional two-dimensional (2D) culture is available as an in vitro experimental model. However, the culture system reportedly does not recapitulate the in vivo cancer microenvironment. We recently developed a tissueoid cell culture system using Cellbed, which resembles the loose connective tissue in living organisms. The present study performed 2D and three-dimensional (3D) culture using prostate and bladder cancer cell lines and a comprehensive metabolome analysis. Compared to 3D, the 2D culture had significantly lower levels of most metabolites. The 3D culture system did not impair mitochondrial function in the cancer cells and produce energy through the mitochondria simultaneously with aerobic glycolysis. Conversely, ATP production, biomass (nucleotides, amino acids, lipids and NADPH) synthesis and redox balance maintenance were conducted in 3D culture. In contrast, in 2D culture, biomass production was delayed due to the suppression of metabolic activity. The 3D metabolome analysis using the tissueoid cell culture system capable of in vivo cancer cell culture yielded results consistent with previously reported cancer metabolism theories. This system is expected to be an essential experimental tool in a wide range of cancer research fields, especially in preclinical stages while transitioning from in vitro to in vivo.

## 1. Introduction

Cancer research, such as the development of anticancer drugs, requires the development of feasible experimental models of organisms that are easy-to-use and cost-effective. At present, a conventional easy-to-use in vitro experimental model of a two-dimensional (2D) culture method is available. However, most studies utilizing this experimental method have failed to demonstrate sufficient safety and efficacy of test drugs in the preclinical stage [1,2,3,4,5,6]. Approximately 11% (only 5% in the field of oncology) of in vivo drug resistance studies using the 2D culture system reach the final phase of demonstrating sufficient efficacy and safety [7], whereas those using the three-dimensional (3D) culture system appear to better simulate the organism’s environment [8]. 3D culture systems using spheroids, extracellular matrices (e.g., collagen), systems for generating cancer organoids and cancer-tissue-originated spheroid methods have recently been reported, showing the fast development of 3D culture [9]. Scaffolds play an important role in the formation of 3D structures. Although there are various types of scaffold materials available, we recently developed a tissueoid cell culture system [10] using a 3D culture scaffold Cellbed^TM^ (Japan Vilene, Tokyo, Japan) that consists of highly pure silicate fiber. Previous 3D culture studies of various cancer cell lines, such as colorectal cancer, Barrett′s adenocarcinoma, gastroesophageal junction cancer, gastric cancer and tongue cancer, have shown that 3D culture systems allow good morphological observations resembling in vivo conditions [10]. Cellbed is an approximately 200-µm-thick ultrafine nonwoven fiber scaffold [11]. The structure of Cellbed is similar to that of loose connective tissue in the organism. Cancer cells change their morphology and move through the pores with a mean diameter of 7–8 µm, which is measured by the bubble point and a mean flow pore test. Compared to the conventional 3D culture systems, the 3D culture system of Cellbed has the following advantages: (I) the absence of other biogenic components, which allows the exclusive study of cancer cells; (II) ease of studying cancer cells similar to 2D culture systems; (III) long-term culture capability; (IV) chemical stability; and (V) 3D culture mounted with a mounting medium with a refraction index equivalent to that of a silicate fiber, which leads to the transparency of Cellbed, allowing postculture observation of the cells with a light microscope [10,11].

In the current study, prostate cancer cell lines (LNCaP and PC-3) and bladder cancer cell lines (UMUC3 and RT-112) were cultured in a 2D culture system and a 3D culture system using Cellbed. Prostate cancer is the second most common cancer in the world [12]. Overall, 80% of prostate cancer is localized cancer with a good prognosis. In contrast, the prognosis of metastatic prostate cancer is poor, with a 5-year survival rate of 30% [13]. Since prostate cancer is sensitive to testosterone, patients with prostate cancer usually undergo hormone therapy. However, they gradually become resistant to it [13]. Bladder cancer is the seventh most common cancer in the world [12]. The most common histological type of bladder cancer is urothelial carcinoma, accounting for 90% of all cancer incidence. Overall, 70–80% of all cancer incidence is identified as noninvasive carcinoma at the first visit, with 15–20% becoming invasive [12]. Patients with unresectable or metastatic bladder cancer usually undergo anticancer treatment. However, they have a poor prognosis with a median survival of 14 months [14]. Chemotherapy is used for the treatment of these cancers in an advanced stage. In this study, metabolomic analysis was performed to examine the differences in metabolites due to differences in culture conditions after conventional 2D or novel 3D culture using the tissueoid cell culture system.

## 2. Results

### 2.1. Hematoxylin and Eosin (H&E) Staining

**Figure 1:**
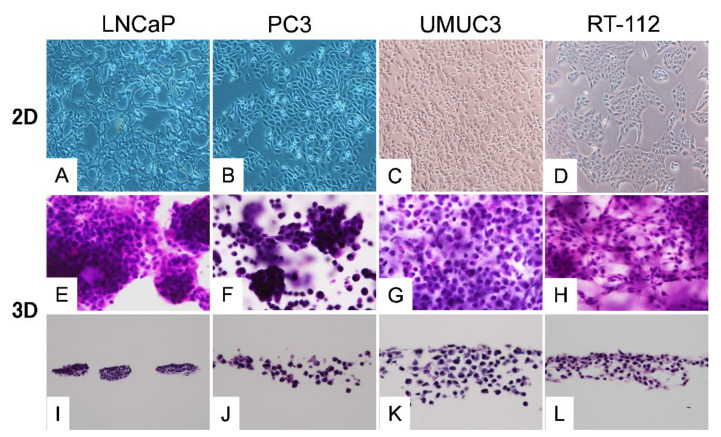
Histology of each cell line. **A**–**D**: Two-dimensional (2D) culture. **E**–**H**: The cells cultured in three-dimensional (3D) culture for 2 weeks were directly HE-stained together with Cellbed. **I**–**L**: The vertical cross-sections of the Cellbed sheets. In 2D, all cell lines showed planar growth (**A**–**D**). In 3D, highly differentiated LNCaP cells showed nodular growth (**E**,**I**), whereas PC-3 cells with poor differentiation showed a mixture of cancer cells that formed small agglomerates and those that existed alone (**F**,**J**). UMUC3 cells with poor differentiation showed solitary but invasive growth (**G**,**K**). RT-112 cells showed loose binding and spindle shape (**H**,**L**).

In the 2D culture, the planar growth of cancer cells was observed, whereas in the 3D culture, cell-specific differentiation was morphologically observed (Figure 1). In prostate cancer cells, highly differentiated LNCaP cells showed nodular growth, whereas PC-3 cells with poor differentiation showed a mixture of cancer cells that formed small agglomerates and those that exist alone. In bladder cancer cells, most UMUC3 cells were solitary. Spindle-shaped RT-112 cells were infiltrating.

### 2.2. Metabolomic Analysis

**Figure 2:**
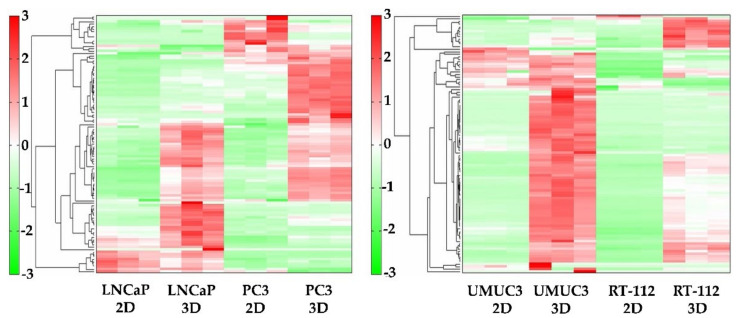
Cluster analysis and heatmap of metabolomic analysis. The levels of most metabolites in all cell lines were higher in 3D than in 2D.

**Figure 3:**
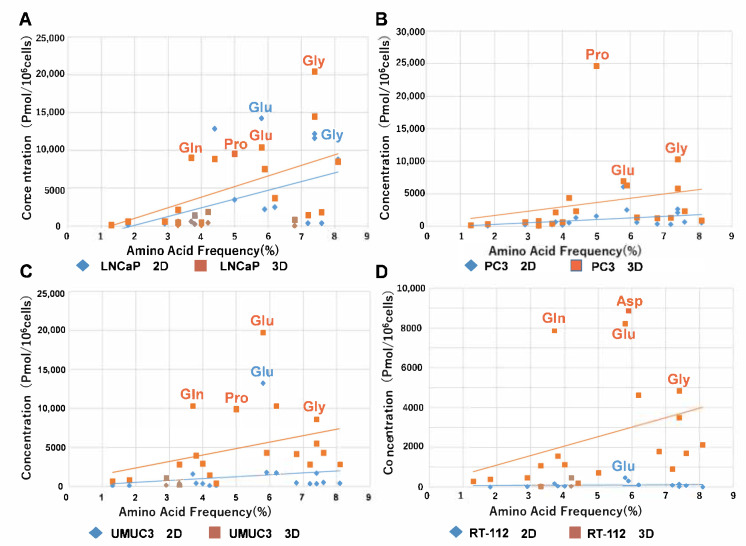
Comparison of the concentration of free amino acids in each cancer cell between 2D and 3D. (**A**) LNCaP, (**B**) PC3, (**C**) UMUC3, (**D**) RT-112. The y-axis represents the respective levels of free 20 amino acids in four cell lines in 2D and 3D in the metabolomic analysis. The x-axis represents the average levels of free amino acids in noncancerous mammal cells reported in the previous literature [15]. The slopes of the approximate lines which plotted free amino acids in all cell lines were larger in 3D than in 2D in all cell lines. These findings suggested that cancer cells in 3D produced many more amino acids than those in 2D.

The heatmap of metabolomic analysis showed lower levels of metabolites in 2D than in 3D for all cell lines (Figure 2). The free amino acid production levels were substantially higher in 3D than in 2D (Figure 3). In a plot with the x-axis as average free amino acid levels in noncancerous mammal cells [15] and the y-axis as the amino acid levels in the metabolomic analysis, the slope of the approximate line tended to be higher in 3D than in 2D (Figure 3). In all cell lines, the concentrations of glutamic acid and glycine were higher than those of other free amino acids. The concentration of glutamine was high in LNCaP, UMUC3 and RT112 cells (Figure 3A,C,D). The concentration of Asp was high in RT112 cells (Figure 3D), whereas the concentration of Pro was high in LNCaP, PC-3 and UMUC3 cells (Figure 3A–C).

**Figure 4:**
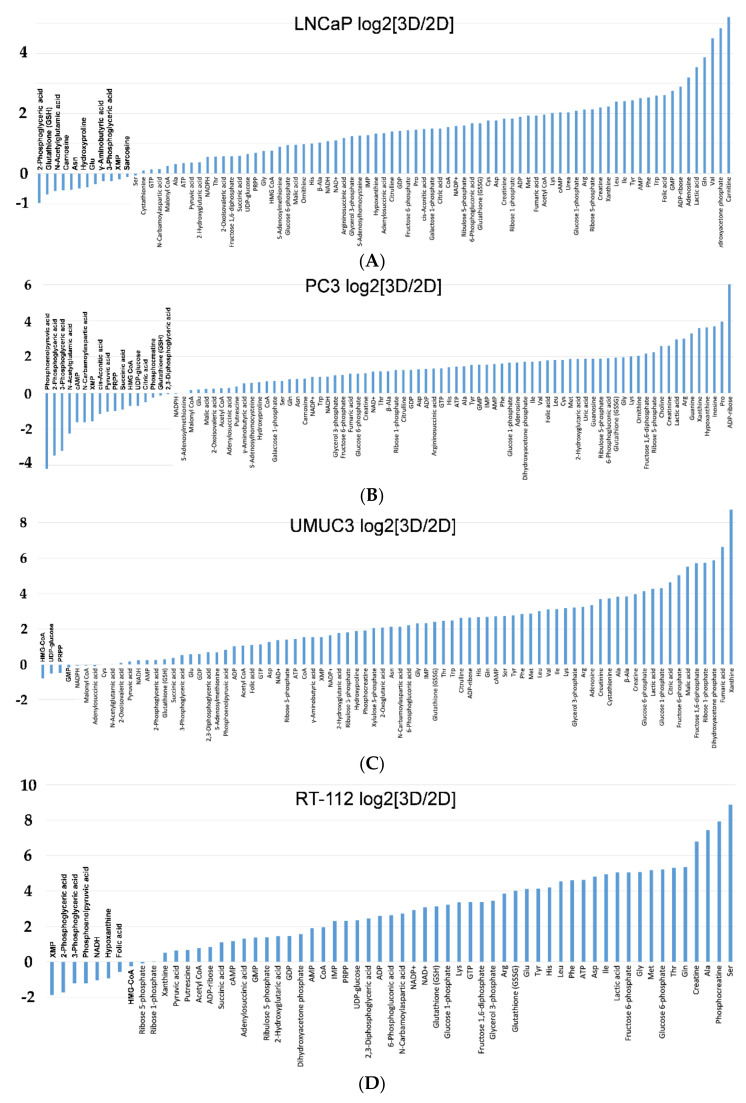
(**A**) Logarithmic plots of LNCaP cells in 3D/2D. The levels of metabolites in the downstream of glycolysis, intermediates of nucleic acid metabolism and most of the essential amino acids were higher in 3D than in 2D. The levels of some of the amino acids, the metabolites of glycolysis, GSH, etc. were lower in 3D than in 2D. (**B**) Logarithmic plots of PC-3 cells in 3D/2D. The levels of most amino acids, the intermediates of nucleic acid metabolism, GSSG, etc. were higher in 3D than in 2D. The levels of metabolites in the downstream of glycolysis and TCA intermediates were lower in 3D than in 2D. (**C**) Logarithmic plots of UMUC3 cells in 3D/2D. The levels of most metabolites and amino acids in the upstream of glycolysis were higher in 3D than in 2D. The levels of metabolites and intermediates of nucleic acid metabolism in the downstream of glycolysis were lower in 3D than in 2D. (**D**) Logarithmic plots of RT-112 cells in 3D/2D. The levels of metabolites in the upstream of glycolysis and amino acids were higher in 3D than in 2D. The levels of metabolites in the downstream of glycolysis and the intermediates of nucleic acid metabolism were lower in 3D than in 2D.

Analysis of each metabolite level in all cancer cells using logarithmic plots of 3D/2D showed a higher tendency of most amino acids in the upstream of glycolysis in 3D than in 2D and a lower tendency of metabolites in the downstream of glycolysis in 3D than in 2D (Figure 4A–D).

#### 2.2.1. Glycolysis

**Figure 5:**
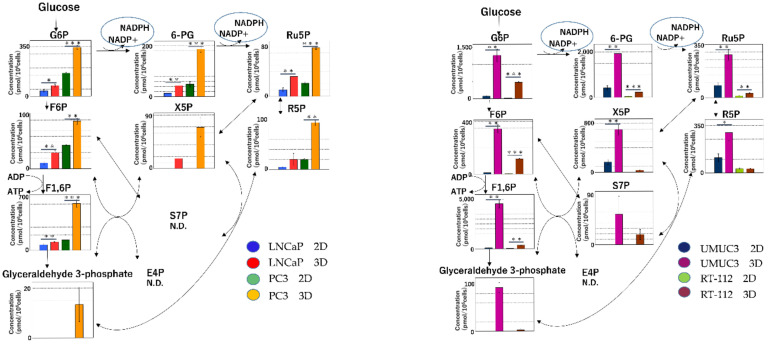
Glycolysis and redox balance in two-dimensional (2D) and 3D culture. Purine, a nucleotide, is biosynthesized using ribose 5-phosphate, which is generated in the pentose phosphate pathway (PPP). The levels of the intermediates of glycolysis were significantly lower in 2D than in 3D. (* *p* < 0.05, ** *p* < 0.01, *** *p* < 0.001).

The metabolite levels of G6P, F6P, F1, 6P and glyceraldehyde-3-phosphate, which are intermediates of glycolysis, were significantly lower in 2D than in 3D (Figure 5). 

#### 2.2.2. Maintenance of Redox Balance

**Figure 6:**
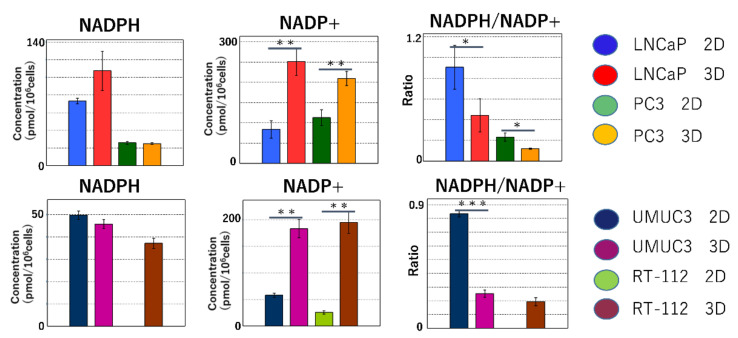
Redox balance in two-dimensional (2D) and 3D cultures. NADPH is primarily produced in the oxidative processes of the PPP. The reducing effect of NADPH is important in ROS scavenging as well as in lipid and amino acid syntheses. In the cancer cell lines (LNCaP, PC-3 and UMC3), the NADPH/NADP+ ratio is significantly lower in 3D than in 2D. These findings indicate that NADPH was consumed for ROS elimination and biomass biosynthesis. (* *p* < 0.05, ** *p* < 0.01, *** *p* < 0.001).

NADPH is mainly produced in the pentose phosphate pathway (PPP). NADPH protects cells from oxidative stress by reduction. The reduction of NADPH plays an important role in lipid and amino acid synthesis. In the cancer cell lines (LNCaP, PC-3 and UMC3), the significantly lower NADPH/NADP+ ratio in 3D than in 2D indicates that NADPH is used in reactive oxygen species (ROS) scavenging and the biosynthesis of biomass (Figure 6).

#### 2.2.3. Metabolomic Analysis of the Tricarboxylic Acid (TCA) Cycle and Oxidative Phosphorylation in Mitochondria

**Figure 7:**
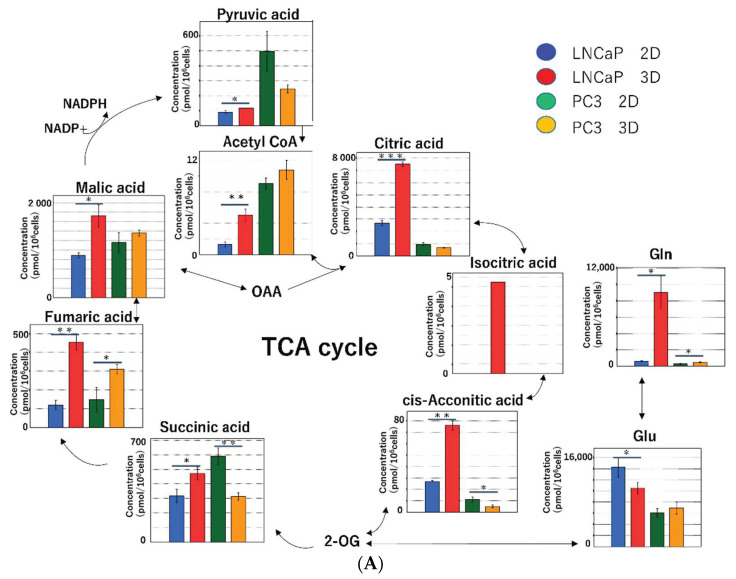

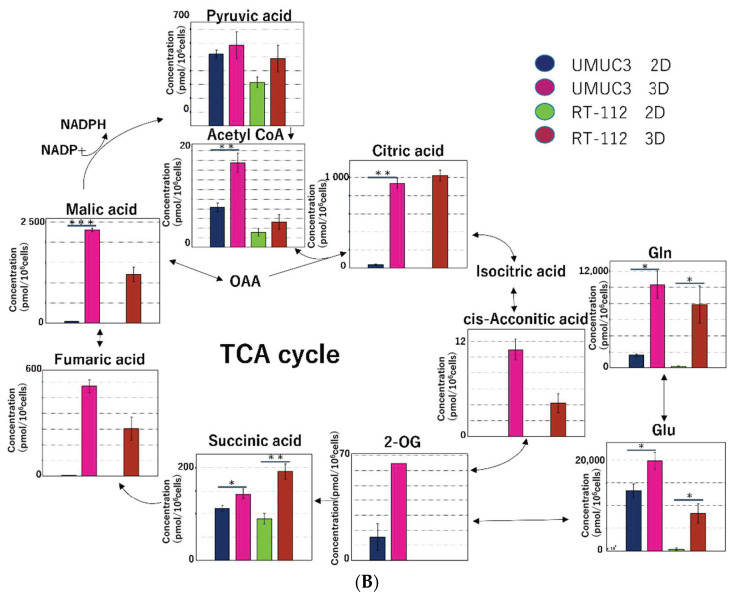
(**A**) Metabolomic analysis of the TCA cycle in the prostate cancer cell lines (LNCaP and PC-3). In LNCaP cells, the levels of most metabolites were higher in 3D than in 2D. In PC-3 cells, the levels of pyruvic, succinic and citric acids were higher in 2D than in 3D. (**B**) Metabolomic analysis of the TCA cycle in the bladder cancer cell lines (UMUC3 and RT-112). In both UMUC3 and RT-112 cells, the levels of metabolites were higher in 3D than in 2D. (* *p* < 0.05, ** *p* < 0.01, *** *p* < 0.001).

**Figure 8:**
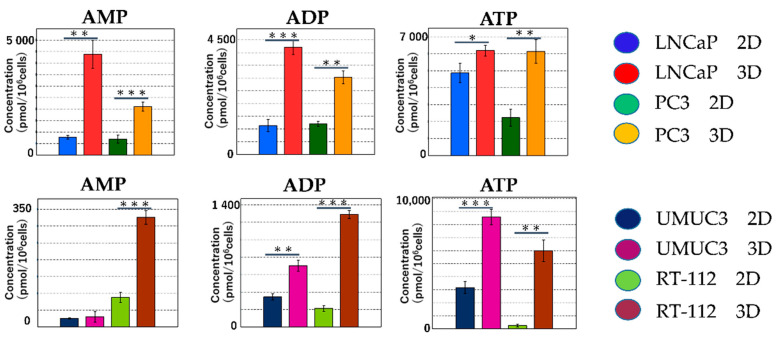
The levels of ATP, ADP and AMP in all cell lines were significantly higher in 3D than in 2D. (* *p* < 0.05, ** *p* < 0.01, *** *p* < 0.001).

The metabolite levels in the TCA cycle were higher in 3D than in 2D. The difference between 2D and 3D was greater for fumaric acid and maleic acid (Figure 7A,B). The main function of mitochondria is ATP production. Following the redox reactions in the TCA cycle (i.e., NADH into NAD+; FAD into FADH2), ATP is produced by consuming oxygen in the electron transport system. During the process, radical oxygen species (ROS) are generated [16]. The production levels of ATP/ADP/AMP were significantly higher in 3D than in 2D (Figure 8). 

## 3. Discussion

In this study, we performed the 2D and 3D culture of bladder and prostate cancer cell lines. Morphological comparison of cells in 2D and 3D by H&E staining of culture cells in Cellbed showed cell-specific morphological differentiation in 3D culture cells. In addition, metabolomic analysis of metabolites in 2D and 3D showed significantly higher levels of most metabolites in 3D than in 2D.

In normal cells, oxidative phosphorylation in mitochondria plays a major role in energy production. However, most cancer cells produce energy by aerobic glycolysis due to impaired mitochondrial function, even in the presence of sufficient oxygen [17,18]. This is called the Warburg effect. However, Warburg′s idea of “impaired mitochondrial function in cancer cells” was incorrect. In fact, it has become evident that cancer cells produce most ATP by oxidative phosphorylation in mitochondria [19,20]. Therefore, mitochondrial metabolism plays an important role in tumor growth and carcinogenesis [21,22,23]. Although aerobic glycolysis seems to be an inefficient method of ATP production, cancer cells have metabolic demands exceeding ATP production [24]. The division and growth of cancer cells require a large biomass, such as amino acids, nucleotides (nucleic acids), lipids and NADPH [24,25,26].

There have been few studies comparing 2D and 3D culture using metabolomic analysis. In a previous study on tongue cancer cells, we compared 2D and 3D culture with Cellbed and xenograft using metabolomic analysis and found a considerable difference in metabolites between 2D and 3D and a similarity in metabolites between 3D and xenograft [27]. In this study, we reported that the cancer cells in the 3D culture and xenografts were shown to actively conduct glycolysis and the citric acid cycle, using nutrients like glucose for biomass biosynthesis and ATP production by oxidative phosphorylation, and also to maintain the redox balance. Conversely, the results from the 2D system suggest that a unique form of metabolism was used to enable cell survival under conditions different from the in vivo environment. Furthermore, a large amount of essential and nonessential amino acids was biosynthesized in 3D. The quantity of amino acids produced was low in the 2D culture, whereas that in the 3D culture was high. The levels of glutamic acid and glutamine were particularly high in 3D compared to those in 2D [27]. Glutamine is an oxidative substrate for the TCA cycle and a starting material for the synthesis of macromolecules [19]. The amide nitrogen of glutamine is used for the synthesis of nucleotides and amino acids, whereas glutamine-derived carbon is used for the synthesis of glutathione, amino acids and lipids. Increased glutaminolysis (glutamine catabolism) has been found in various types of tumors [16]. In the present study, the levels of glutamic acid and glycine were higher than those of other amino acids in all cell lines, whereas the levels of glutamine were high in LNCaP, RT-112 and UMUC3 cells. The concentration of Asp was high in RT-112 cells, whereas the concentration of Pro was high in LNCaP, PC-3 and UMUC3 cells. A previous study on bladder cancer cells using metabolomic analysis reported higher levels of Pro, alanine and Asp. These amino acids play an important role in the synthesis of the extracellular matrix [28].

In the 3D culture, the activation of the PPP led to an increase in the biosynthesis of nucleotides. The starting material of purine nucleotides is ribulose 5-phosphate generated in the PPP. Inosinic acid is converted into AMP and GMP, which in turn are converted to ATP and GTP for use in nucleic acid synthesis. Pyrimidine nucleotides are synthesized from carbamoyl phosphate and aspartic acid. Synthesized orotic acid is combined with phosphoribosyl diphosphate to produce orotidylic acid, which in turn is converted to UMP (due to decarboxylation) and then into CTP and TTP. The pyrimidine base is decomposed, metabolized and further decomposed into carbon dioxide in the TCA cycle. Both purine and pyrimidine bases require nitrogen derived from glutamine, aspartic acid and glycine [29]. However, the high levels of amino acids in 3D suggest that these amino acids may be used in the biosynthesis of nucleic acid. NADPH functions as a donor with strong reducing power for many ROS detoxification enzymes to neutralize [30]. Cancer cells in oxidative stress activate oxidative PPP that produces NADPH, following the increase in the ROS levels, leading to the production of a large amount of NADPH. In the present study, the NADPH/NADP^+^ ratio was significantly lower in 3D than in 2D in LNCaP, RT112 and UMUC3 cells, suggesting that the NADPH produced in oxidative PPP is used to neutralize ROS.

Cell culture studies are essential for the elucidation of cancer pathophysiology and therapeutic exploration. 2D culture systems are commonly used in cell culture experiments. However, unlike the structure of the organisms in the 2D culture system, cells in organisms show 3D growth. Although drug development ranging from clinical trials to marketing requires time and funding, more than 92% of new drug candidates are discarded during clinical trials [31]. Some studies have reported differences in drug sensitivity between 2D- and 3D-cultured cells. A study on ovarian cancer cell lines (OV-MZ-6 and SKOV-3) showed a marked difference in the efficacy of anticancer drugs doxorubicin and paclitaxel between 3D cancer cells cultured with hydrogel and 2D-cultured cells, a 40–60% decrease in cancer cells after the addition of paclitaxel and an 80% decrease in 2D-cultured cancer cells [32]. In addition, a study of sensitivity to anticancer drugs using 3D-cultured colorectal cancer cell lines (Lovo), breast cancer cell lines (MCF-7) and lung adenocarcinoma cell lines (PC-3) showed a higher resistance of Lovo and MCF-7 cells to CDDP, 5-FU and ADM in 3D than in 2D [33]. Furthermore, the addition of cytotoxic sodium selenite to lung cancer (A549) and pancreatic cancer (PANC1) cells in magnetic 3D culture showed a higher resistance to sodium selenite in 3D than in 2D [34]. Further examination using metabolomic analysis showed (i) a substantially lower decrease in the levels of metabolites in glycolysis and the TCA cycle in 3D (spheroid) than in 2D due to the addition of sodium selenite and (ii) lesser impairment of GLU and GSH metabolism in 3D than in 2D [34].

A limitation of our 3D culture method is the absence of blood flow and the interstitium, unlike in an in vivo environment. Therefore, it remains challenging to perform the 3D culture of fibroblasts and vascular endothelial cells in Cellbed. Nonetheless, it is to some extent possible to simulate the interstitium by coating Cellbed with collagen, laminin and fibronectin [10]. The function of 3D-cultured rat hepatocytes grown in Cellbed and coated with these lasts longer in 3D than in 2D [35]. This is believed to result from the addition of an extracellular matrix facilitating the maintenance of the 3D cellular structure and intercellular interactions. In addition, Cellbed coated with type IV collagen, in which tongue cancer cells HSC-3 and HSC-4 were 3D-cultured, was found to be more susceptible to invasion [10].

In conclusion, this study demonstrated that 3D culture promotes the biosynthesis of biomass, ATP production by oxidative phosphorylation and the maintenance of redox balance by actively using glycolysis and the TCA cycle by way of nutrients, such as glucose. Metabolomic analysis of 3D culture using the tissueoid cell culture system that mimics the morphology and function of cancer tissues in an organism showed results consistent with theories and previous studies on cancer metabolism. The system is useful for various areas of cancer research such as screening for the development of new anticancer drugs and may be an essential experimental tool that plays an important role in the preclinical stage (from in vitro to in vivo).

## 4. Materials and Methods

### 4.1. Cancer Cells and Cell Culture

Two types of prostate cancer cell lines (LNCaP and PC-3) and two types of bladder cancer cell lines (UMUC3 and RT-112) were used in this study. Prostate cancer cell lines were obtained from the American Type Culture Collection (ATCC, Manassas, VA, USA). LNCaP is an androgen-dependent cancer cell line derived from lymphatic metastatic tumors in a male patient with prostate cancer [36]. PC-3 cells are poorly differentiated androgen-independent cancer cells derived from lumbar metastatic tumors in a male patient with prostate cancer [37]. UMUC3 was collected from primary tumors in a male patient with G2 bladder urothelial carcinoma [38]. RT-112 was collected from primary tumors in a female patient with G2 bladder urothelial carcinoma. Prostate cancer cells were cultured in the RPMI1640 medium (Nacalai Tesque, Kyoto, Japan) supplemented with 10% FBS and 1% ABAM. Bladder cancer cells were cultured in the DMEM medium (Nacalai Tesque) supplemented with 10% FBS (Sigma-Aldrich, St. Louis, MO, USA) and 1% ABAM (Invitrogen, Carlsbad, CA, USA). Both 2D and 3D cultures were incubated at 37 °C and 5% CO_2_. 3D culture was performed as reported in our previous study [27]. The following is a summary of the procedure: First, the cells were seeded at a concentration of 4 × 10^4^ cells/mL in a 12-well plate on which Cellbed with a diameter of 19 mm and 1 mL medium had been placed previously. The cells in Cellbed were transferred to a plate with a larger base area, specifically, a 6 cm dish with 6 mL medium, 7 days after grafting. Because the medium tends to accumulate in the center of Cellbed, the plates were shaken manually at regular intervals, specifically, once every 2–3 days, to prevent a lack of nutrients in cancer cells due to local accumulation of nutrient-depleted medium.

### 4.2. H&E Staining

Cells cultured in a 10 cm dish in the 2D culture system were examined and imaged with a light microscope. Two weeks after seeding in Cellbed, the cells were washed with phosphate-buffered saline. We performed H&E staining directly together with Cellbed. Furthermore, after a 30-min fixation in 10% formalin and paraffin embedding of multiple divided Cellbed sheets, the 3D structure of the cells was examined via H&E staining of the vertical and horizontal cross-sections of the Cellbed sheets.

### 4.3. Metabolomic Analysis

Cells were cultured in a 10 cm dish in the 2D culture system. The cells were seeded at an expected concentration of 1 × 10^6^ cells/dish 3 days after seeding. Using MTT assay, the cells in the 3D culture system were seeded at an expected concentration of 1 × 10^6^ cells per three Cellbed sheets 2 weeks after seeding. Following our request, Human Metabolome Technologies Inc. (Tsuruoka, Japan) performed metabolomic analysis of cryogenically treated specimens with an equal number of cells for both 2D and 3D culture. We analyzed 116 kinds, especially water-soluble and ionic metabolites related to central energy metabolism (glycolysis, TCA cycle, amino acid metabolism and nucleic acid metabolism), with high sensitivity and high accuracy.

### 4.4. Analysis Methods

Amino acid levels of each cell line in 2D and 3D cultures were compared in a plot with the x-axis as the average amino acid levels in mammal cells and the y-axis as the amino acid levels in the metabolomic analysis. In addition, logarithmic plots of each metabolite in 3D/2D were created to compare metabolite levels in both 2D and 3D cultures. The values in the logarithmic plots were calculated with 2 as the base of the logarithm.

### 4.5. Statistical Analysis

The statistical analysis for the metabolome analysis was undertaken using Welch′s *t* test (* *p* < 0.05, ** *p* < 0.01, *** *p* < 0.001).

## Data Availability

Data sharing is not applicable to this article.
